# Developmental genetics of the *C. elegans *pharyngeal neurons NSML and NSMR

**DOI:** 10.1186/1471-213X-8-38

**Published:** 2008-04-09

**Authors:** Claes Axäng, Manish Rauthan, David H Hall, Marc Pilon

**Affiliations:** 1 Dept. of Chemical and Biological Engineering, Chalmers University, Box 462, S-405 30, Göteborg Sweden; 2 Dept. Neuroscience, Albert Einstein College of Medicine, Bronx, New York, NY10461, USA; 3 Dept. Cell and Molecular Biology, Göteborg University, Box 462, S-405 30, Sweden

## Abstract

**Background:**

We are interested in understanding how the twenty neurons of the *C. elegans *pharynx develop in an intricate yet reproducible way within the narrow confines of the embryonic pharyngeal primordium. To complement an earlier study of the pharyngeal M2 motorneurons, we have now examined the effect of almost forty mutations on the morphology of a bilateral pair of pharyngeal neurosecretory-motor neurons, the NSMs.

**Results:**

A careful description of the NSM morphology led to the discovery of a third, hitherto unreported process originating from the NSM cell body and that is likely to play a proprioceptive function. We found that the three NSM processes are differently sensitive to mutations. The major dorsal branch was most sensitive to mutations that affect growth cone guidance and function (e.g. *unc-6, unc-34, unc-73)*, while the major sub-ventral branch was more sensitive to mutations that affect components of the extracellular matrix (e.g. *sdn-1*). Of the tested mutations, only *unc-101*, which affects an adaptin, caused the loss of the newly described thin minor process. The major processes developed synaptic branches post-embryonically, and these exhibited activity-dependent plasticity.

**Conclusion:**

By studying the effects of nearly forty different mutations we have learned that the different NSM processes require different genes for their proper guidance and use both growth cone dependent and growth cone independent mechanisms for establishing their proper trajectories. The two major NSM processes develop in a growth cone dependent manner, although the sub-ventral process relies more on substrate adhesion. The minor process also uses growth cones but uniquely develops using a mechanism that depends on the clathrin adaptor molecule UNC-101. Together with the guidance of the M2 neuron, this is the second case of a pharyngeal neuron establishing one of its processes using an unexpected mechanism.

## Background

The development of organ innervation requires numerous axon guidance cues that are evolutionarily conserved and more easily studied in simple organisms. Research in this area has led to a general model where receptors on growth cones, which are motile structures at the tips of growing axons, respond to a wide variety of cues that include long-range signals such as netrins or secreted forms of semaphorins, or contact-dependent cues that include ephrins, Slit and many components of the extracellular matrix [1-3]. Different growth cones may express different receptors and therefore respond to different molecular cues as they explore their environment. Growth cones migrate towards attractants and away from repellents, and in this way establish unique axon trajectories. This model has great explanatory power and undoubtedly is generally correct. However it poses a conundrum: the diversity of neuron types and trajectories seems vast, yet the known guidance signals and receptors are few. In an effort to explain how the limited guidance toolbox can generate a wide variety of neuron trajectories, and also in the hope of discovering novel mechanisms of axon guidance, we are pursuing an in-depth explanation of how the pharyngeal neurons develop in *C. elegans*.

The *C. elegans *pharynx is an isolated neuro-muscular organ composed of only 80 cells. It is located at the anterior end of the worm where its primary function is to rhythmically pump and grind bacterial suspensions (food) before transferring the product to the intestine. Twenty neurons innervate the pharynx in a highly reproducible fashion, and most of these may be ablated without severe effects on viability or pharyngeal pumping [4]. There are few interactions between the pharynx and other somatic tissues, either during morphogenesis or during its physiological function.

The pharyngeal nervous system can be divided into four principal parts: two subventral nerve cords, one dorsal nerve cord, and one circular pharyngeal nerve ring located within the posterior half of the metacorpus [5]. One interesting aspect of the system is that the neurons extend trajectories within muscle cell folds, rather than between a basement membrane and hypodermal cells, as do most body neurons. This unique topology may rely on hitherto uncharacterized mechanisms for guiding axonal development. In a published study we have previously described a genetic analysis of the axon guidance of the bilaterally symmetrical motorneurons M2L and M2R that are located within pharynx of *C. elegans *[6]. In that study we discovered that the M2 neurons establish most of their trajectories in a growth cone-independent manner, a finding that raised the question of whether other pharyngeal neurons also utilize such mechanisms.

To address this question and better understand how the pharyngeal nervous system develops, we studied the genetic pathways that regulate guidance of another pair of pharyngeal neurons, the NSMs (Pharyngeal **n**euro**s**ecretory-**m**otor neurons), that can be visualized using the *pTPH-1::GFP *reporter [7]. The cell bodies of the NSML and NSMR serotonergic neurons are located sub-ventrally in the pharyngeal metacorpus, and each extends posteriorly one bifurcating major process and one thin minor process ([5]; this article). The NSMs have neurohumoral functions and were postulated to sense bacteria in the pharyngeal lumen by their proprioceptive endings and to communicate with the rest of the worm's body via secretion into the pseudocoelomic fluid [5]. Ablation of the NSMs significantly decreases the enhanced slowing response that starved worms exhibit upon encountering food, suggesting that they contribute to this behavior [8]. Our study shows that different branches of the same neuron respond differently to the same set of guidance cues. In particular, the minor (dendritic) process of the NSM establishes its trajectory in a manner that is dependent on the clathrin adaptor complex, whereas the major (axonal) process requires more typical growth cone and guidance molecules for its proper guidance and branching.

## Results and Discussion

### Expression profiling of the *pTPH-1::GFP* reporter

**Figure 1:**
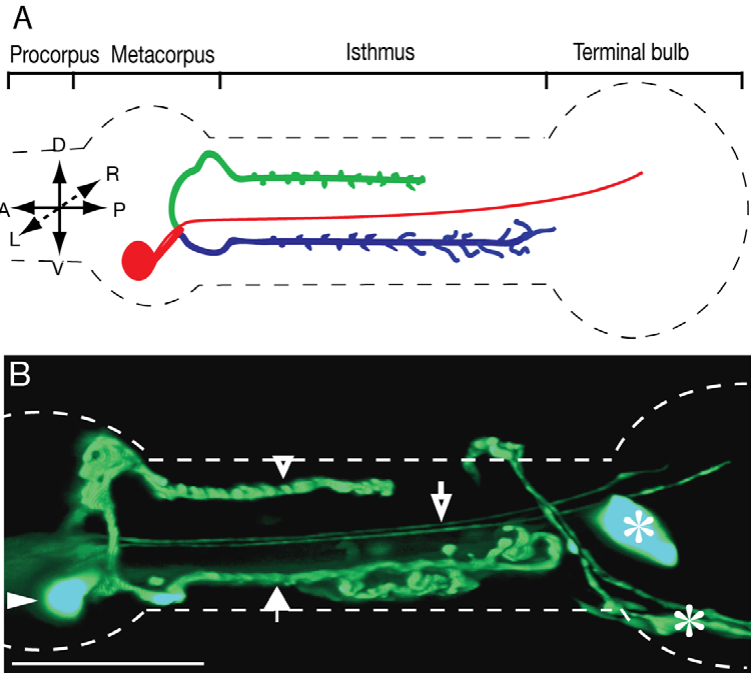
**Morphology of the NSM neurons**. (A) Cartoon of one NSM neuron, NSML (B) Flattened stack of confocal images of an adult worm expressing *pTPH-1::GFP*. The main structures shown in panels A and B are the cell body (red circle; filled triangle), the major sub-ventral (blue; filled arrow) and dorsal (green; open triangle) and the minor (red line; open arrow) processes. The scale bar represents 20 μm.

**Figure 2:**
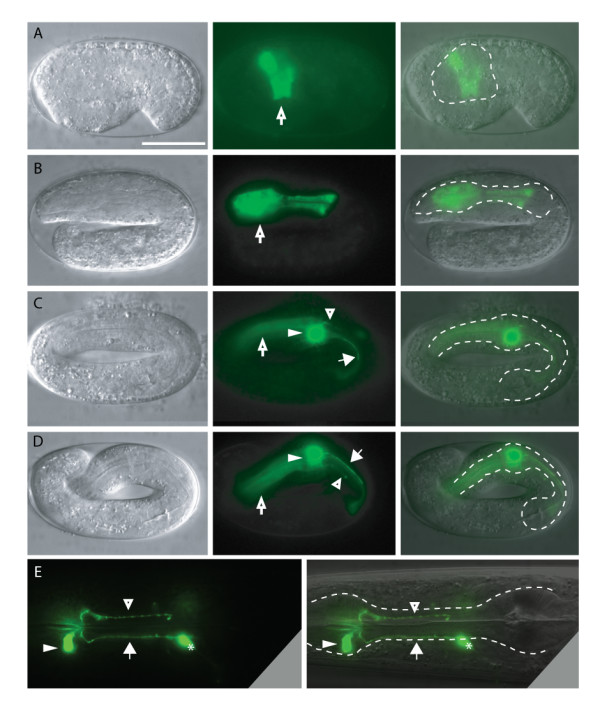
**Time course of expression of the *pTPH-1::GFP *reporter**. Transgenic worms were photographed at different developmental stages: (A) ~330 minutes-old embryo; (B) Two-fold stage; (C) Two-and-a-half-fold stage; (D) Three-fold stage; and (E) Young adult. The following GFP-positive cells or structures are indicated: pharyngeal muscle (open arrows), NSM cell body (filled triangle), and the major sub-ventral (filled arrow) and dorsal (open triangle) processes. The minor process is not visible in these images. Scale bar represents 20 μm.

Pharyngeal expression of the *pTPH-1::GFP *reporter is limited to the NSM neurons in larvae and adults; outside the pharynx, expression is also seen in the nerve ring and some other extra-pharyngeal neurons e.g. HSN, ADF (Fig. 1). During embryogenesis, expression of *pTPH-1::GFP *within the pharyngeal primordium can first be observed at approximately 300 min. of development (Fig. 2). When the pharyngeal primordium begins to elongate at two-fold stage, strong expression of the *pTPH-1::GFP *reporter is observed both in the muscle cells of the procorpus and metacorpus. In the late three-fold stage, as the pharynx elongates and matures, pharyngeal expression of the *pTPH-1::GFP *reporter becomes restricted to the NSM neurons; pharyngeal muscle expression weakens and eventually disappears completely by the time of hatching [7]. Because of the embryonic expression of *pTPH-1::GFP *in pharyngeal muscles, the NSM processes have already established their trajectories by the time that they can be unambiguously scored using this reporter. It is therefore not possible to directly study the NSM neurons as they are developing using the *pTPH-1::GFP *reporter, although it is useful to examine the final morphology of these neurons. Note that none of the mutants tested in the course of the present study affected the levels of *pTPH-1::GFP *expression (see below).

### Morphology of the NSM neurons

**Figure 3:**
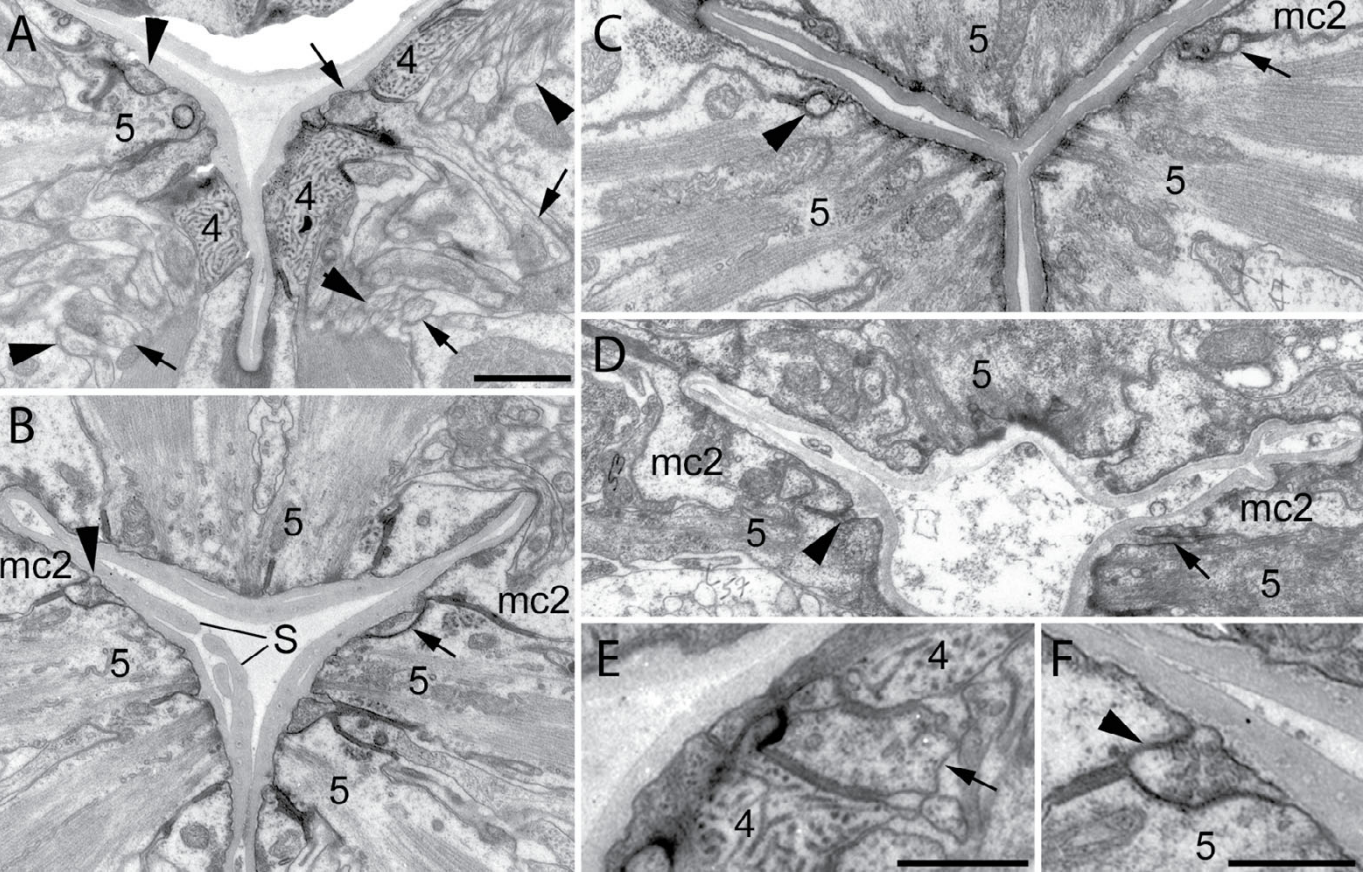
**Thin NSM processes seen in serial thin sections by TEM**. (A) Profiles of NSML (large arrowheads) and NSMR (arrows) in the region of the pharyngeal nerve ring. Each neuron shows several profiles within this section as it travels around the ring, including some profiles that are beyond the edge of the panel. NSMR sends a large process inward from the ring to the cuticle, diving between two portions of the right ventral pharyngeal muscle pm4 (4), to which it is attached by adherens junctions (called desmosomes in [5]; compare to their Figure 9). A few small vesicles are present within NSMR near the cuticle. The thin process of NSML (top left arrowhead) has already migrated laterally, passing underneath pharyngeal muscle pm5 (5), and is also filled with clear vesicles. (B) Less than one micron posterior to panel A, the thin processes of NSML (arrowhead) and NSMR (arrow) are migrating laterally to positions lying between pm5 (5) and the marginal cells (mc2), and both thin processes are filled with clear vesicles. Large adherens junctions secure pm5 to mc2 at their inner borders. The region shown is subject to severe distortion during each pharyngeal pumping cycle, and the inner portion of these cells, together with the NSM processes will move laterally to positions outside the view of this panel with every contraction. Thin cuticle strands extend within the pharyngeal lumen; these are part of the sieve (S). (C) Far posterior to panel B, this section lies about halfway along the pharyngeal isthmus. The minor processes of NSML and NSMR are now thinner and contain few or no vesicles. Each lies within a groove between pm5 and mc2 cells, well secured by the local adherens junctions. Again this portion of the thin process is subject to maximum shearing forces during pharyngeal pumping, and is presumed to be stretch-sensitive. (D) A section at the border of the posterior pharyngeal bulb, where the minor processes of NSML and NSMR are about to end, becoming even thinner and still in close contact to pm5 and mc2 cells and their luminal adherens junctions. This is also the posterior limit of pm5 and mc2 cells. (E) Closeup of NSMR from a section close to panel A, which shows several vesicles restricted to the portion of the process closest to the pharyngeal cuticle. (F) Closeup of NSML from the same section shown in B, to show the clear vesicles and nearby adherens junction just to the left of NSML. All panels were scanned from archival TEM images (negatives or prints) of Albertson and Thomson (1976) stored in the Hall archive [5]. Scale bars: A-D share the same 1 μm scale bar; the scale bars in E and F represent 0.5 μm.

The NSML and NSMR neurons are bilateral homologues. The cell body of each neuron resides within a subventral pm4 muscle cell in the metacorpus and extends one major and one minor process (Fig. 1). The major process bifurcates near the cell body to produce one long subventral process that runs posteriorly within the subventral nerve cord of the isthmus, and a shorter dorsal process running posteriorly in the dorsal nerve cord. To reach the dorsal cord, these processes first pass through the pharyngeal nerve ring. Both dorsal processes then run together posteriorly within the dorsal nerve cord of the isthmus. Both dorsal and subventral processes run near the outside edge of the nerve cords, very close to the pseudocoelom, and make synapses to the pharyngeal basement membrane and muscle cells. Within the isthmus these processes form en passant synaptic swellings and short synaptic branches at the basal zone, contacting the basement membrane to release vesicles into the pseudocoelom. The dorsal process terminates half way through the isthmus whereas the subventral process ends just anterior to the terminal bulb. The minor dendritic process is thin and synapse-free, extending under the cuticle of the pharyngeal lumen within the isthmus. This thin process had not previously been reported as a separate entity in a detailed ultrastructural description of the pharynx [5], but careful examination of archival electron micrographs confirmed their presence and shapes (Fig. 3). The thin process likely plays a proprioceptive function because it is devoid of organelles and is embedded in close contact with the cuticle lining the pharyngeal lumen.

### Morphology of the NSM synaptic branches over time and in response to serotonin

**Figure 4:**
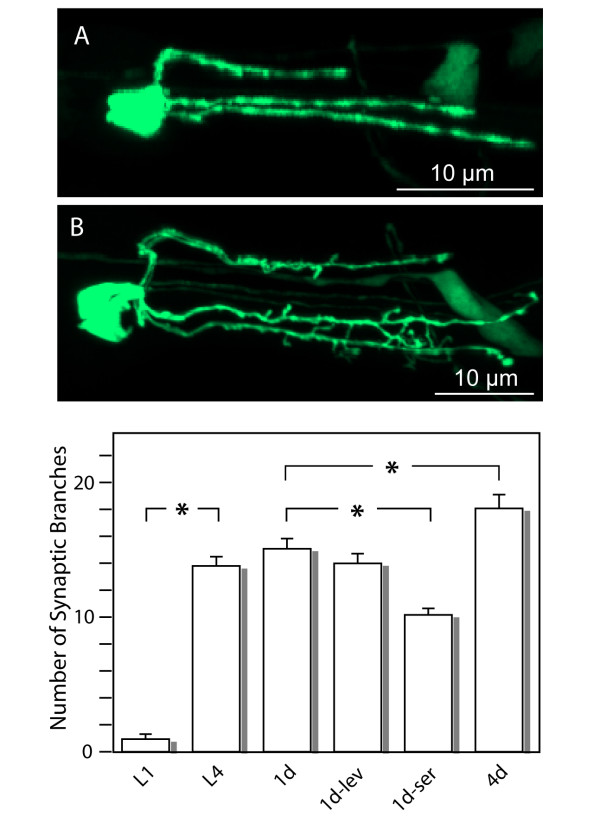
**Development of the NSM synaptic branches**. Flattened confocal stacks are shown for larval stages L1 (A) and L4 (B). Note the prominent synaptic branches at the L4 but not L1 stage. The graph shows the average number of synaptic branches from at least 11 individuals for each age or condition. 1d-lev and 1d-ser refer to animals grown in levamisole or serotonin from the L1 stage, respectively, and scored as 1-day old adults. Asterisks indicate significant differences with p < 0.05 between the indicated groups.

*C. elegans *neurons are typically simple and unbranched [9]. For this reason, it was of particular interest to examine the synaptic branches found on the NSM major processes during development and to investigate whether their number or shapes change in response to activity. We found that synaptic branches develop post-embryonically: they are mostly absent at the L1 stage but are numerous by the L4 stage and continue to increase in number between 1-day and 4-day old adults (Fig. 4). When synaptic swellings were counted in L1 larvae, we found that they are present in numbers similar to the number of synaptic branches in 4-days old adults, namely 18.4 ± 0.7 and 18.1 ± 1.0, respectively. This indicates that it is the morphology of the synaptic branches that changes during development/aging, rather than the number of synapses per se. To test if pharyngeal muscle activity could feedback onto the NSM neurons to regulate the dynamics of the synaptic branches, we grew worms in the presence of 50 μM levamisole from the L1 larval stage to 1-day old adults. Levamisole is an acetylcholine analogue that causes muscles to contract constitutively, thus causing the animals to also grow more slowly. It had no effect on the number of synaptic branches for developmentally age-matched animals, suggesting that there is no feedback between contraction and the number of synaptic branches. We next tested for the possibility that the increase in synaptic branches may be a feedback response to the need for more efficient release of serotonin by the serotonergic NSM neurons. We found that growing worms from L1 to 1-day old adults in the presence of 7.5 mM serotonin caused a significant reduction in the number of synaptic branches (Fig. 4). This result supports the notion that the synaptic branches increase to meet a need for more efficient serotonin secretion into the pseudocoelom, and represent an example of synaptic plasticity in *C. elegans*.

### Estimating the natural variation in NSM morphology

**Figure 5:**
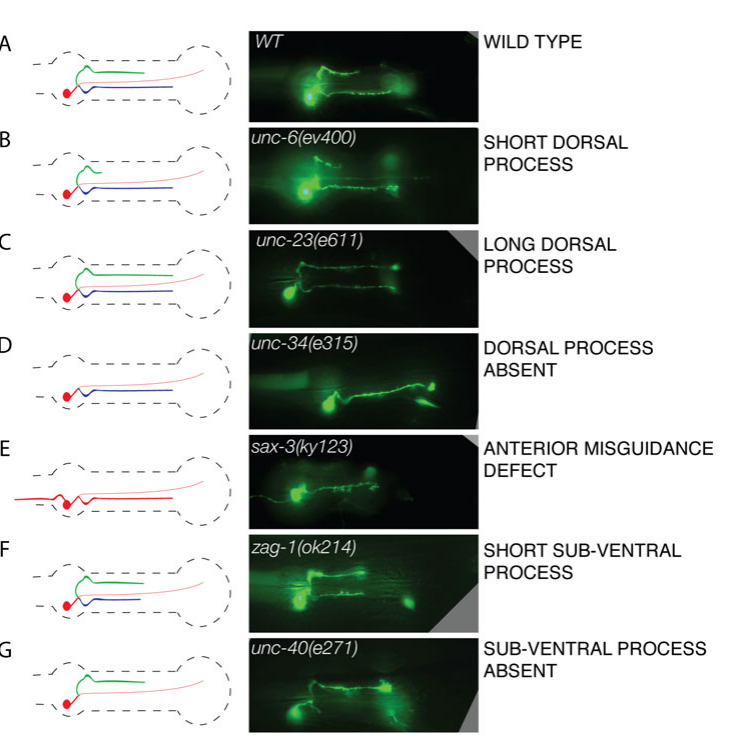
**Main NSM phenotypic categories observed in this study**. The left column shows a cartoon rendition of the type of defect shown in the middle column and given the category name listed in the right column. Color coding is as in Fig. 1.

We have examined the morphology of the NSM neurons in almost forty different genetic backgrounds, and grouped the most commonly observed phenotypes into distinct classes (Fig. 5 and Table 1). In wild-type control worms, more than 95% of the NSM neurons were scored as normal, with only 4% showing a short dorsal process and 1% showing a short sub-ventral process (Table 2). As control mutations that should not have specific effects on the NSM neurons we used *unc-30 *and *unc-46*. These mutations affect differentiation or GABA secretion in GABAergic neurons and should not have direct effects on NSM development since the NSMs are serotonergic neurons. Not surprisingly, the *unc-30 *and *unc-46 *mutations had little effect on the NSMs: 93 and 91% of the NSM neurons were scored as normal in these two mutants, and the rare abnormal neurons were cases where the length of the processes appeared as either too short or too long, compared to the wild-type (Table 2). The average and standard deviation for the three control genetic backgrounds is 93± 2% of NSM neurons being scored as normal. We considered that any mutation that causes defects in more than 15% of the scored NSM neurons (twice as many as in the controls) is having a significant effect.

**Table 1:** List of the mutations used in this study.

**Genotype**	**Allele**	**Protein**	**Pathways**	**Ref**
*eat-4(ky4)*	loss-of-function	glutamate transporter	chemotaxis, feeding behaviour	[61]
*fax-1(gm83)*	null	Nuclear hormone receptor	Axon guidance and morphogenesis	[62]
*kal-1(gb503)*	null	Adhesion-type protein	Neurite branching and morphogenesis	[33]
*mab-20(bx24)*	unknown	Semaphorins	Morphogenesis	[63]
*max-1(cz1632)*	null	Cytoplasmic protein with PH, MyTH4 and FERM domains	Axon guidance	[64]
*mec-1(e1066)*	Likely null	Extracellular EGF and Kunitz domain protein	Localization of receptors on touch neurons	[36]
*mnm-2 (et2)*	hypomorph*	Zn finger transcription factor	Axon guidance	[52]
*mod-5(n3314)*	null	Serotonin transporter	serotonin uptake	[48]
*pha-2(ad472)*	hypomorph	homeodomain protein	pharynx development	[49]
*sax-3(ky123)*	null	Homolog to *Drosophila *ROUNDABOUT	Axon guidance and morphogenesis	[65]
*sdn-1(zh20)*	null	type I transmembrane heparan sulfate proteoglycan	cell-cell and cell-matrix adhesion	[66]
*slt-1(eh15)*	null	Ligand for *Robo *receptor *sax-3*	Axon guidance and morphogenesis	[25]
*Syg-1(ky652)*	null*	novel transmembrane protein	Synaptic specificity in the HSNL	[42]
*tph-1(mg280)*	null	tryptophan hydroxylase	Serotonin synthesis	[7]
*unc-5(e53)*	null	Repellent netrin receptor	Netrin signaling	[21]
*unc-6(ev400)*	null	Netrin-1	Netrin signaling and morphogenesis	[17]
*unc-14(e369)*	nonsense	novel	axon guidance	[67]
*unc-23(e611)*	unknown	unknown	muscle attachment	[68]
*unc-34(e315)*	nonsense*	Signal transduction protein Enabled.	Axon guidance	[69]
*unc-40(e271)*	null	Attractive/repellent, netrin receptor	Netrin signaling and morphogenesis	[70]
*unc-44(e362)*	unknown	Ankyrin	Axon guidance and morphogenesis	[71]
*unc-46(e177)*	nonsense*	Novel transmembrane protein	GABA neurotransmission	[72]
*unc-51(e369)*	nonsense	serine/threonine kinase	axonal elongation	[73]
*unc-52(e1421)*	unknown*	perlecan	muscle structure and growth factor-like signaling pathways	[74]
*unc-61(e228)*	nonsense*	Septin	axonal and distal tip cell migration, and postembryonic cytokinesis	[40]
*unc-69(e587)*	null	Short coil-coil domain containing protein	Axon guidance and presynaptic organization	[75]
*unc-73(e936)*	?**	guanine nucleotide exchange factor	vulva morphogenesis and axonal outgrowth	[10]
*unc-76(e911)*	deletion*	coiled-coil protein	axonal outgrowth and fasciculation	[14]
*unc-86(n846)*	null	transcription factor with a POU-type homeodomain	fate determination and differentiation of several neural lineages	[76]
*unc-101(m1)*	truncated*	adaptin	synapse function	[46]
*unc-104(rh1016)*	unknown?	kinesin-like motor protein	synapse function	[47]
*unc-112(r367)*	missense	pleckstrin homology domain-containing protein	dense body/M-line component	[77]
*unc-115(e2225)*	?**	actin binding LIM protein	axon guidance	[16]
*unc-119(e2498)*	?**	novel	axonal branching and fasciculation	[12]
*unc-129(ev55)*	null	A member of the TGF-beta family of secreted growth factors	Axon guidance and morphogenesis	[31]
*vab-1(dx31)*	null	Tyrosine kinase, EPH (ephrin) receptor family	Axon guidance and morphogenesis	[78]
*vab-8(ev411)*	Hypomorph*	A kinesin-like motor protein	Axon guidance and morphogenesis	[79] [80]
*wrk-1(ok695)*	null	Wrapper/Rega-1/Klingon homolog	Axon guidance and morphogenesis	[81]
*zag-1(zd85)*	nonsense*	Homeobox transcription factor	Axon guidance and morphogenesis	[82]

* *unc-34(315) *is a nonsense mutation believed to produce truncated functional protein. *unc-46(e177) *is a nonsense mutation (W/stop) in the fourth exon leading to a small truncated protein and most likely a null allele. *unc-52(e1421) *is a mutation that alters the splice donor site of exon 16 and reduces or abolishes splicing to the 3' end of exon 16. *unc-61(e228) *is base transition changing a CGA to TGA at position 156 resulting in a truncated protein. zag-1(*zd84*) is a nonsense mutation allele causing complete or nearly complete loss of function. *unc-76(e911) *has an 11 nucleotide deletion in the 6^th ^exon that leads to a frame shift and truncated protein (severe loss of function). *unc-101(m1) *is a mutation at nucleotide 1314, changing a CAA codon to a TAA stop codon. *vab-8(ev411) *is mutation at a splice donor site (G-A).

** *unc-119 (e2498)*: Maduro and Pilgrim (Genetics, 1995) it is a 1.6 kbp insertion (Tc1) in the third coding exon

** *unc-73(e936) *The *e936 *allele changes the splice donor of intron 16 from 5*9 *. . . /**G**TAGGGC . . . 3*9 *to 5*9 *. . . **T**TAGGGC . . . 3*9*. The possibility of alternative splicing has not been carefully examined in any of the *unc-73 *mutant alleles. from the steven et al ref from the table.

** *unc-115(e2225) e2225 *is an insertion of a Tc4 transposable element at the exon 6/intron 6 boundary.

**Table 2:** Effects of mutations on the morphology of the pharyngeal NSM neurons.

**Genotype**	**Normal (%)**	**Short dorsal process (%)**	**Short sub-ventral process (%)**	**Long dorsal process (%)**	**Dorsal process absent (%)**	**Sub-ventral process absent (%)**	**Anterior guided axons (%)**	**Absent Minor process (%)**	Others* (%)	**N**
Controls										

wild-type	95	4	1							185
*unc-30*	93	4		3					1	196
*unc-46*	91	6	1	1	1					328

Growth cone-defective mutants										

*unc-14*	72	11	4		1				10	202
*unc-41*	96	3	1							172
*unc-51*	23	22	16		29	1		1	9	226
*unc-73*	30	20	6	4	27	10			3	283
*unc-76*	35	37	2	1	25				1	272
*unc-115*	87	1	2	10					1	225
*unc-119*	29	41	4	1	22				4	269

Positional cue and cue interpretation mutants.										

*fax-1*	84	8	6	2						198
*kal-1*	61	10	15	1	6	5			2	219
*max-1*	95	1	1	3						228
*mob-20*	100									194
*sax-3*	26	1	3	2	12		37		19	210
*slt-1*	41	16	22	7	4	6			4	247
*smp-2*	64	18	6	5	5	3				168
*unc-5*	40	26	6	3	13	11			1	181
*unc-6*	40	24	1	2	32				1	307
*unc-34*	9	7		3	77	1	1		3	204
*unc-40*	39	3	5	12	26	8	4		4	229
*unc-69*	26	38		8	28	1				184
*unc-129*	57	15	9	7	6	6				198
*vab-1*	87	7		3	1	1			1	181
*vab-8*	95			2	1				2	214
*wrk-1*	83	10	3		2	2				189
*zag-1*	83	2	4	4	5	2			1	200
*zig-4*	92		4	4						100

Extracellular matrix protein										

*mec-1*	75		4	8		9			4	253
*sax-7*	32	1	4	37	7	30			1	145
*sdn-1*	41	11	5	4	6	32			1	254
*unc-23*	68	3	2	5	2	1			20	237
*unc-52*	64	8	7	3	2				16	213
*unc-61*	75	5		16	3	1				172
*unc-112*	75	6	9	7	3	1				340

Synapse Function mutants										

*syg-1*	43	26	1		24	5			1	183
*unc-101*	0	4	2	7	3	5		100	13	223
*unc-104*	94	5	1							174

Genes important for proper function of NSM										

*mod-5*	73	17	7		3	1				168
*tph-1*	94	1	3	2						168

Pharyngeal morphology mutants										

*mnm-5*	79	2		1	8		1		11	267
*pha-2*	4	1	1		21		45		27	141

*The category "others" reflect specific phenotypes encountered for individual mutants, as follows:

***mnm-5***: misplaced cell bodies, complete loss of guidance.

***pha-2***: misplaced cell bodies; sub-branching of the misguided branches; minor process absent; multiple branching.

***sax-3***: thickened endings of axons; misplaced cell bodies; multiple branching; all branches are anterior misguided; sub-branching of the misguided branches; minor process absent.

***unc-23***: thickened endings of axons.

***unc-51***: milder defects similar to *unc-73 *(ectopic branching).

***unc-52***: thickened endings of axons.

***unc-73***: thickened endings of axons; multiple branching from cell bodies.

***unc-101***: thickened endings of axons; multiple branching from cell bodies.

### The major NSM process develops in a growth cone dependent manner

Several mutations known to impair growth cone function, e.g. *unc-51, unc-73*, *unc-76 *and *unc-119*, caused defects in the major NSM process and affected both the long subventral and, usually more severely, the short dorsal branch. In particular, both branches of the major process are often either absent or show premature termination in the *unc-73 *mutant, which affects a guanine nucleotide exchange factor involved in Rac signaling within growth-cones [10]. In this mutant, 27% of the dorsal processes and 10% of the sub-ventral processes are absent. *unc-119 *encodes a novel protein important for regulating axon branching and fasciculation [11,12]. The mutant exhibited a high frequency of defects in the dorsal branch which was missing in 22% and too short in 41% of cases.

In the *unc-51 *mutant background only the dorsal process is markedly affected. *unc-51 *encodes a Serine/threonine-protein kinase involved in membrane recycling within growth cones [13]. In the mutant, 29% of the NSM neurons lack a dorsal process while 38% have defects in the termination of the dorsal or subventral process along the anterior-posterior axis, indicating that *unc-51 *is important both for initiation of the dorsal processes and for their proper termination (Table 1). *unc-76*, which encodes a FEZ protein (fasciculation and elongation protein [14,15], displays a similar phenotype. Other growth cone defective mutants such as *unc-14 *and *unc-115 *also show defects in the dorsal process but at a lower frequency. *unc-14 *is suggested to be a regulator of *unc-51 *in axonal elongation and guidance [13], whereas *unc-115 *encodes an actin-binding LIM Zn-finger protein [16].

### Netrin signalling guides the major NSM processes

*unc-6 *encodes a *C. elegans *netrin ligand that guides many axons along the dorso-ventral axis [17,18]. It can act as an attractant or a repellent, depending on type of receptor that is expressed on the growth cone. *unc-40 *encodes a netrin transmembrane receptor important for dorso-ventral guidance [19] and also determines the polarity of neurons [20], whereas the *unc-5 *co-receptor is required for netrin-mediated repulsion [21]. *unc-6 *is also important for anterior-posterior guidance [20,22]. In the *unc-6 *mutant background, we found that 32% of the NSM neurons have no dorsal process and 24% have a short dorsal process, suggesting that *unc-6 *guides the dorsal process in establishing its trajectories and in determining its lengths. Mutation in the *unc-5 *gene mainly results in premature termination of both the dorsal and sub-ventral process of the NSM neurons: 26% of the dorsal processes are short and 13% are absent. In *unc-40 *mutants, 26% of the dorsal and 8% of the sub-ventral processes are absent. Interestingly, 4% of the NSM neurons show anterior misguidance defects, indicating that *unc-40 *gene is not only important for proper guidance of the major process but also in determining the polarity of the NSM neurons.

### *sax-3* determines the axonal polarity of the NSM neurons independently of *slt-1*

Slit-Robo signaling is involved in midline guidance in both invertebrate and vertebrates [23]. In *C. elegans*, mutations in the ROBO homolog *sax-3 *cause cell migration and midline guidance defects [24]. In *sax-3 *mutants the NSM neurons often project one axon branch anteriorly (37%) or exhibit a morphology so abnormal, with cell bodies frequently misplaced, that it is not easily classified (19%). 12% of the NSM neurons in these mutants lack the dorsal processes and 3% have a short sub-ventral process. These results indicate that *sax-3 *is important in establishing the anterior-posterior polarity of the NSM neurons, or their correct positioning within the primordium, and for axon initiation.

*slt-1 *is the only known ligand for *sax-3 *in *C. elegans *and is involved in dorsal-ventral and midline guidance [25]. The *slt-1 *mutant caused the dorsal and sub-ventral and processes to be short in 16% and 22% of cases, respectively. The fact that the *sax-3 *mutation caused more severe and qualitatively different defects than the *slt-1 *mutation indicates that it can bind at least one other ligand during NSM neuron development.

### Other guidance genes necessary for the major NSM processes

*unc-34 *is homologous to the signal transduction protein ENABLED that is required for axon guidance and plays a vital role in downstream signalling for both the *netrin *and *slit *pathways [26]. In *unc-34 *mutants, 77% of the dorsal processes are absent indicating that this gene is essential for the initiation of the dorsal process from the NSM cell body. Another guidance gene, *unc-69 *is also important for the proper development of the major process. *unc-69 *encodes a protein with a short coiled-coil domain that is generally important for axon outgrowth and proper synaptic formation [27,28]. In *unc-69 *mutants, the initiation (28%) and termination (38%) of the dorsal process are primarily affected, with similar weaker effects on the sub-ventral process.

The ephrins are a highly conserved family of proteins that are involved in axon guidance in vertebrates whereas in *C. elegans *they seem to play only a minor role in axon guidance but have critical functions in multiple aspects of epidermal morphogenesis [29,30]. To find out if ephrins are involved in NSM guidance, we scored the NSMs in mutants for the sole ephrin receptor *(vab-1)*. Our results indicate that ephrins play only a minor role, if any, in the development of the dorsal processes of the NSM neurons: *vab-1 *mutant exhibit 7% small dorsal process, 1% missing dorsal process and no sub-ventral defects.

Another important axon guidance molecule in *C. elegans *is *unc-129*, which encodes a member of the TGF-beta family of secreted growth factor signalling molecules that acts as a dorso-ventral guidance cue [31,32]. The dorsal and subventral processes of the NSM neurons showed a variety of initiation and termination defects that affected 43% of NSM neurons.

Finally, *kal-1*, which encodes an extracellular protein with a fibronectin III domain that likely acts as a guidance cue [33], had a slightly stronger effect on the sub-ventral processes (20% absent or short) than on the dorsal one (16% absent or short).

### Extracellular matrix (ECM) molecules and the major NSM process

ECM molecules provide instructive signals for many neurons in *C. elegans*, and are also important also for the maintenance of their morphologies [34]. We studied the NSM trajectories in the *mec-1, sax-7, sdn-1, unc-23, unc-52 *and *unc-112 *mutants. Our findings indicate that ECM or ECM-adhesion genes are involved in both the initiation and the termination of the major NSM processes. *sdn-1 *is a type I transmembrane heparan sulfate proteoglycan thought to be involved in cell-cell or cell-matrix adhesion [35]. Of all the mutations studied, *sdn-1 *caused the most severe defects on the sub-ventral process, with 32% of them being absent, and another 5% being too short. The large transmembrane ECM adhesion molecule *sax-7 *caused severe defects in both the long dorsal and the subventral processes, with 37% and 34% being affected, respectively. *mec-1 *and *unc-112 *encode two extracellular proteins that are also involved mainly in development of the sub-ventral process. MEC-1, is an ECM protein with multiple EGF and Kunitz domains and is involved in organizing channel complexes within mechanosensory assemblies [36], and UNC-112 is a membrane-associated structural/signalling protein that has been implicated in linking the actin cytoskeleton to the ECM [37]. In *mec-1 *mutants, 8% of the NSM sub-ventral processes are absent and another 4% are short. In *unc-112 *mutants, 9% of the NSM sub-ventral processes are short. Other ECM genes such as *unc-23, unc-52 *exhibit low penetrance defects in the major process of the NSM neurons. Both these mutants lead to initiation and termination problems in the dorsal and sub-ventral processes of the NSM, which often caused thickened endings. *unc-61 *encodes a *C. elegans *septin that is thought to regulate the contact points between the cytoskeleton and the cell cortex, in this way possibly influencing attachment between the cell and the extracellular matrix [38-40]. In the *unc-61 *mutant, 19% of the dorsal processes were too long or absent, and 5% of the subventral branches were truncated.

### Synapses are essential for the normal development of the NSM major process

Some *C. elegans *neurons require proper localization of active synapses for their morphology and maintenance [41]. We found that the trajectories of the dorsal NSM process is often defective in the *syg-1 *mutant, but that both of the major processes are generally not affected by mutations in *unc-101 *or *unc-104*. SYG-1 is an immunoglobulin super-family protein that determines the position of specific synapses in *C. elegans *[42,43]. 26% of NSM neurons in *syg-1 *mutants have a shorter dorsal process and 24% have no dorsal process, indicating that formation of synapses is essential both for the initiation and the proper guidance of the dorsal process. The weak effects of *unc-101 *and *unc-104 *on the major processes perhaps reflect their intracellular functions [44-47]. For example, while the *unc-104 *mutant does have reduction in the formation of synapses, its function seems to reside principally in synaptic vesicle trafficking rather than synapse formation per se [45,47].

### NSM function mutations

The NSM neurons are serotonergic, and their physiological activity depends on the *tph-1 *and *mod-5 *genes that respectively encode a tryptophane hydroxylase and a serotonin transporter [7,48]. Short dorsal (17%) and subventral (7%) processes were found in the *mod-5 *mutant. This result supports the idea that functioning synapses may play a role in the development or maintenance of the NSM neurons.

### Proper cell positioning is required for determining the polarity of the NSM processes

Unsurprisingly, we found that the NSM processes depend on correct cellular context to establish their trajectories: the NSM neurons were grossly abnormal in the pharyngeal mutant *pha-2*. *pha-2 *encodes a homeobox transcription factor necessary for the successful differentiation of the pm5 muscle cells that make up the isthmus, and also for the establishment of the correct morphologies of several pharyngeal structures, including the isthmus and metacorpus [49,50]. In *pha-2 *mutants, the isthmus is short and thick due to misplaced cell nuclei. In these mutants, 45% of NSM neurons abnormally send a major process anteriorly, and 21% of the dorsal processes are absent; another 27% of the neurons show other defects such as multiple branching and misplaced cell bodies. Our results indicate that *pha-2 *is not only required for the proper positioning of the NSM cell bodies within the pharynx but also determines the axonal polarity of the NSM neurons. *mnm-5 *is a mutant in which the cell bodies of the pharyngeal M2 neurons are often misplaced into the metacorpus, rather than being located in their normal position within the posterior bulb [6]. The molecular identity of *mnm-5 *is currently unknown. In this mutant, 8% of the NSM dorsal processes are missing, and 11% of the NSM cell bodies are misplaced or misshaped, and send grossly misguided branches.

### Development of the NSM minor process depends on unc-101

**Figure 6:**
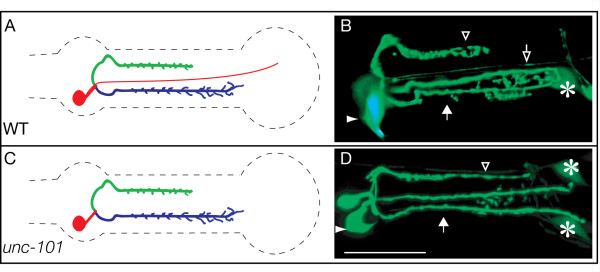
**The thin NSM process is absent in *unc-101 *mutants**. (A-B) and (C-D) show a cartoon summary and flattened stacks of confocal images of an adult wild-type and *unc-101 *worm, respectively. Note that the minor process (open arrow) is present in wild-type but absent in the mutant. The major sub-ventral (filled arrow) and dorsal (open triangle) processes are also shown. The asterisks indicate the ADF cells that lie just outside the pharynx.

Our study has focused mostly on the major processes because the minor process was very difficult to score reliably due to its weak GFP signal. In spite of this, we found that the thin process is rather resistant to mutations that affect growth cones, although low penetrance defects such as occasional truncations are observed in these mutants, suggesting that some growth cone function is directly or indirectly required for its elongation. Interestingly, the NSM minor process was uniquely sensitive to the *unc-101 *mutation. Neurons are polarized cells that contain distinct sets of proteins in their axons and dendrites and the polarized movement of proteins is a result of selective transport. In *C. elegans*, clathrin-associated proteins are known to determine the polarity between axons and dendrites. *unc-101 *encodes a mu1 subunit of the AP-1 clathrin adaptor complex that is involved in polarized dendrite transport and for determining the axon-dendrite polarity in olfactory neurons [44,46,51]. In *unc-101 *mutants, the NSM neurons have no minor process whereas the major process is completely normal (Fig. 6). This result suggests that *unc-101 *is essential for polarized transport of proteins that are important for initiation and development of the minor process of the NSM neuron. Together with the proximal trajectory of the M2 neurons, which is established without the use of a growth cone [6], this is the second case of a pharyngeal neuron establishing part of its arborization through an unexpected mechanism. It may be that the NSM minor process is established by the stretching of the axon between the NSM cell body and a substrate that moves away during development, as is the case for the M2 axon [6,52] and the dendrites of some sensory neurons [53]. This would explain the resilience of the minor process to growth cone mutations. Alternatively, and given that the trajectory of the minor process follows precisely the pm5/mc border (Fig. 3), it is possible that a physically confined adhesion pathway accounts for the developmental robustness of this minor process. While dendrites and axons utilize many of the same guidance cues and mechanisms [54,55], significant differences in their development have previously been noted. For example, dendrites in *Drosophila *are more critically dependent on the secretory pathway during their elongation [56], a finding that echoes the role of *unc-101 *during the development of the NSM dendrite.

### Closing remarks

**Figure 7:**
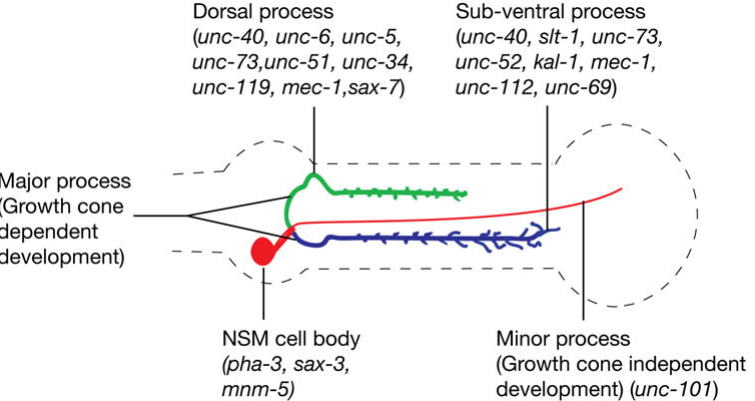
**Summary of the effects of mutations on NSM morphology**. Genes that caused at least 10% defect in each part of the NSM neurons are indicated.

By studying the effects of nearly forty different mutations we have learned that the different processes of the NSM neurons require different genes for their proper guidance and use growth cone dependent mechanisms for establishing their trajectories (summarized in Fig. 7). We also found that the NSM minor process is unique in that its development depends on the clathrin adaptor molecule UNC-101.

One obvious limitation of our work is that we do not know if the tested mutations have a direct or secondary effect on the NSM neurons. We also did not test for cell-autonomous versus non-autonomous functions. Another limitation in our study was that the *pTPH-1::GFP *reporter that we used allowed us only to examine the NSM neurons in their final, or near final configurations. It is possible, although unlikely, that some observed phenotypes were due to the degeneration of neurons that had successfully developed prior to observation. Despite these limitations, it was reassuring that genes acting within a distinct biochemical pathway, i.e. the various classes shown in Table 2, caused similar NSM defects. This allows us to draw specific conclusions regarding the development of the NSM neurons, and broad conclusions regarding the development of nervous systems in general.

In a broad perspective, and also taking into account our earlier work on the guidance of the pharyngeal neuron M2 [6,52], several conclusions emerge from our genetic analyses that are relevant to all developing nervous systems. One is that neuronal process outgrowth is extremely robust: i) with the exception of the effect of *unc-101 *on the NSM minor process, which is a highly unusual branch type, no mutation completely impaired the development of a particular axon; and ii) almost all of the tested axon guidance mutants could perturb the establishment of the studied axon trajectories. The various axons must each be capable of responding to multiple, redundant cues to different effect in order to initiate and guide its unique trajectory. Thus M2 and NSM both depend on many of the same guidance pathways (e.g. *unc-6, slt-1*), yet produce very different morphologies. We have previously shown that the M2 growth cone requires an interaction with the M3 neuron for its proper programming [52]. Each cell must reprogram its growth cone in specific ways according to its cell identity, often multiple times over the course of its migration. Such growth cone reprogramming has been amply documented elsewhere, as in the developing grasshopper limb when the Ti1 pioneer axon contacts specific guidepost cells then proceeds to the next target [57,58], or when commissural neurons cross the midline in *Drosophila *and their growth cones are reprogrammed to prevent them from crossing again [59]. The diversity of axon pathways apparently results not from an immense diversity of molecular cues, but from differences in the relative timing or strength of a growth cone's responses and choice(s) among a limited number of cues. Conclusions more specific to NSM development are presented below.

## Conclusion

1. The serotonergic NSM neurons harbour short synaptic branches that develop post-embryonically, and whose number can be regulated by serotonin levels.

2. The growth of the NSM processes, just like that of the M2 neuron previously studied [6], relies on multiple guidance cues, as well as on growth cone dependent and independent mechanisms.

3. Of thirty-nine mutations tested, only ten produced less than 15% abnormal NSM neurons, yet only three caused highly penetrant defects in more than 80% of NSM neurons (*pha-*2, *unc-34*, and unc-*101*). Of these, only *unc-34 *and *unc-101 *are likely to directly affect the growth of the NSM processes, rather than its guidance. Thus NSM development integrates multiple (redundant) pathways and is a robust mechanism usually capable of withstanding failure in any one pathway.

4. Of the twenty-nine mutants with significant effects on the NSM neurons, twenty-five mutants had more severe effects on the major dorsal than the subventral NSM process, including mutations that impair growth cone function (e.g. *unc-51, unc-73, unc-76*) or guidance cues and their interpretation (e.g. *unc-6, unc-34)*. Thus the dorsal process probably requires more complex guidance decisions relative to the sub-ventral process, as one would expect from its more intricate trajectory.

5. Among the mutations studied, only four (*kal-1*, *sdn-1, slt-1*, and *mec-1) *had more severe effects on the major sub-ventral branch. Of these, *sdn-1 *had the most severe effect, suggesting that adhesion or substrate choice is a relatively important parameter influencing the growth of that branch.

6. Only one mutation, *unc-101*, strongly affected the minor NSM process. Given that UNC-101 is an adaptin molecule orthologous to the mu1-I subunit of adaptor protein complex 1 (AP-1), this suggests that the minor branch does not depend on growth cones for its establishment, but rather relies on the targeted deposition of material from clathrin-coated vesicles or on the clathrin-dependent endocytosis of a signal from the environment by the outgrowing dendrite.

## Methods

### Nematode strains and cultivation

All strains were maintained using standard methods [60], and were grown at 20°C, unless otherwise stated. List of mutants and transgenic strains used in this study:

Linkage group (LG) I: *kal-1(gb503), mod-5(mg280), mab-20 (bx24), unc-14(e57), unc-73(e936), unc-40(e271), unc-101(m1).*

LG II: *tph-1 (mg280), unc-52(e1421), unc-104(rh1016), vab-1(dx31).*

LG III: *eat-4(ky5), mnm-5(et5), unc-69(e587), unc-119(e2498).*

LG IV: *efn-2(ev658), pha-3(ad607), sax-7(ok1244), unc-5(e35), unc-30(e318), unc-44(e362), unc-129(ev55), zag-1(ok214), zdls13.*

LG V: *max-1 (cz1632), mec-1(e1066), unc-23(e611), unc-46 (e177), unc-51 (e369), unc-61 (e228), unc-76 (e911), unc-112 (r367), vab-8(ev411).*

LG X: *fax-1(gm83), pha-2 (ad472), sax-3 (ky123), sdn-1(zh20), slt-1 (eh15), syg-1(Ky652), tag-53 (gk163), unc-115(e222), wrk-1(ok695), zig-4 (gk34).*

*zdls13 *carries the *tph-1::gfp *transcriptional reporter expressed in the NSM, HSN and ADF neurons, and was a gift from Scott Clark. This is strictly a promoter reporter expressing no part of the TPH-1 protein.

### Phenotype scoring and documentation

Worms carrying *zdls13 *were mounted on 2% agarose pads containing a drop of 100 mM levamisole. NSM pharyngeal neuron trajectories were scored at 400× and 1 000× magnification using a Leica DML or a Zeiss Axioplan compound microscope equipped with FITC filter sets, used with excitation 450–490 nm. Each NSM neuron of any given animal was scored into the following categories: Wild-type (cell body is properly placed in metacorpus and both the major and the minor processes show no guidance defect), short-dorsal process (the dorsal process terminates prematurly in the isthmus), short-subventral process (the length of the subventral process is the same or shorter than the dorsal process), long-dorsal process (the dorsal process is as long as the subventral process), no-dorsal process (dorsal process does not extend posteriorly), no-subventral process (the subventral process is absent), anterior-misguidance (one of the processes extends anteriorly within the procorpus instead of growing posteriorly), no-minor process (the minor process is absent) and others (includes defects that are not categorized). Each strain was scored independently by two of the authors and the scoring results were found to be similar and therefore pooled in Table 2.

### Confocal microscopy

Worms were mounted on dried 2% agarose pads (in H2O), paralyzed by one drop of levamisole (100 mM) and examined by a Zeiss LSM 510 META System connected to an inverted Zeiss Axiovert 200 microscope.

### Levamisole and serotonin treatments

For some experiments L1 larvae were grown in the presence of 50 mM levamisole or 7.5 mM serotonin until they became 1-day old adults. They were then mounted on agarose pads, imaged by confocal microscopy and the number of synaptic branches counted by analysing Z-stacks of the images.

### Electron microscopy

Archival prints of the wild type adult pharynx were examined from four serially sectioned animals from the MRC collection (JSA, N2W, N2T and N2U) and several others in the Hall collection to follow the trajectory of the minor dendritic branch and of the multiple short synaptic branches of the two major axons.

## Authors' contributions

CA and MR contributed equally, carried out most of the experiments and helped with the writing of the manuscript. DHH provided electron microscopy results, and helped writing the manuscript. MP helped in experimental design and interpretations, and took the main responsibility for writing the article.
